# From shadows to light: navigating the rare complication of cerebral air embolism from a case report

**DOI:** 10.3389/fmed.2026.1825716

**Published:** 2026-05-20

**Authors:** Ying Zhu, Guanghao Liao, Liu Yang, Han Duan, Qi Zhu, Huang Fang

**Affiliations:** 1 Department of Neurology, General Hospital of Central Theater Command of People’s Liberation Army Graduate Joint Training Base, School of Medicine, Wuhan University of Science and Technology, Wuhan, China; 2 Department of Neurology, General Hospital of Central Theater Command of People’s Liberation Army, Wuhan, China

**Keywords:** case report, cerebral air embolism, cerebral infarction, hyperbaric oxygen therapy, lung biopsy

## Abstract

We report a female patient who underwent computed tomography (CT)-guided percutaneous lung biopsy due to a right lung mass. After the procedure, she developed impaired consciousness. Non-contrast cranial CT revealed small intracranial air bubbles. Diffusion-weighted magnetic resonance imaging showed multiple acute cerebral infarctions. Whole-brain angiography did not reveal any major vessel occlusion. She was diagnosed as iatrogenic cerebral air embolism. After admission, she received intensive treatment including 100% oxygen inhalation and hyperbaric oxygen therapy. Eventually, she regained consciousness and was discharged successfully. Cerebral air embolism is a rare but severe complication of percutaneous lung biopsy with a generally poor prognosis. Clinicians should maintain a high index of suspicion for this condition in patients presenting with acute neurological deficits after lung biopsy. Hyperbaric oxygen therapy demonstrates significant neuroprotective effects and improves clinical outcomes.

## 1 Introduction

Computed tomography (CT)-guided percutaneous lung biopsy is a crucial minimally invasive technique for diagnosing pulmonary space-occupying lesions. However, the procedure may rarely cause a potentially fatal complication—cerebral air embolism (1). Air embolism occurs when air rapidly enters the circulatory system, forming gas bubbles that obstruct blood vessels. Based on the site of embolism, it can be classified into venous air embolism and arterial air embolism. When air enters the arterial circulation and occludes cerebral arteries, it leads to cerebral air embolism, which can cause a series of neurological impairments and is a potentially life-threatening condition (2). Latrogenic cerebral air embolism has an abrupt onset, with clinical symptoms typically appearing during or within minutes to hours after interventional operations such as CT-guided lung biopsy, bronchoscopy, or pre-thoracoscopic artificial pneumothorax. Although its clinical presentation is variable, it is characterized primarily by acute and severe neurological deficits. The most common symptoms include sudden disturbance of consciousness, epileptic seizures, and focal neurological deficits such as hemiplegia and aphasia (3). We herein report a case of cerebral air embolism following percutaneous lung biopsy and review relevant literature to discuss strategies for early diagnosis and management.

## 2 Case report

### 2.1 Clinical data

The patient is a 71-year-old female admitted to our hospital due to “vomiting and impaired consciousness for over 5 h.” She had undergone a CT-guided percutaneous biopsy for a right lung mass at another hospital. En route back to her ward after the procedure, she experienced sudden vomiting, followed by loss of consciousness, unresponsiveness and urinary incontinence, prompting emergency transfer to our hospital. Her past medical history includes hypertension (on amlodipine, blood pressure well-controlled), type 2 diabetes mellitus (diet-and exercise-controlled, no medication), and untreated hyperlipidemia. She denies any allergies, blood transfusion, or major trauma, and is a nonsmoker and non-drinker. She has one son and reached menopause at around age 50. Her spouse and son are in good health and there is no family history of genetic disorders. On admission, physical examination revealed a comatose patient, unable to cooperate with the examination. Both eyes showed conjugate gaze deviation to the left. The left limbs exhibited spontaneous movement. Right upper limb muscle strength was grade 0, and right lower limb muscle strength was grade 2. The National Institutes of Health Stroke Scale (NIHSS) score was 29.

### 2.2 Auxiliary examinations

**FIGURE 1:**
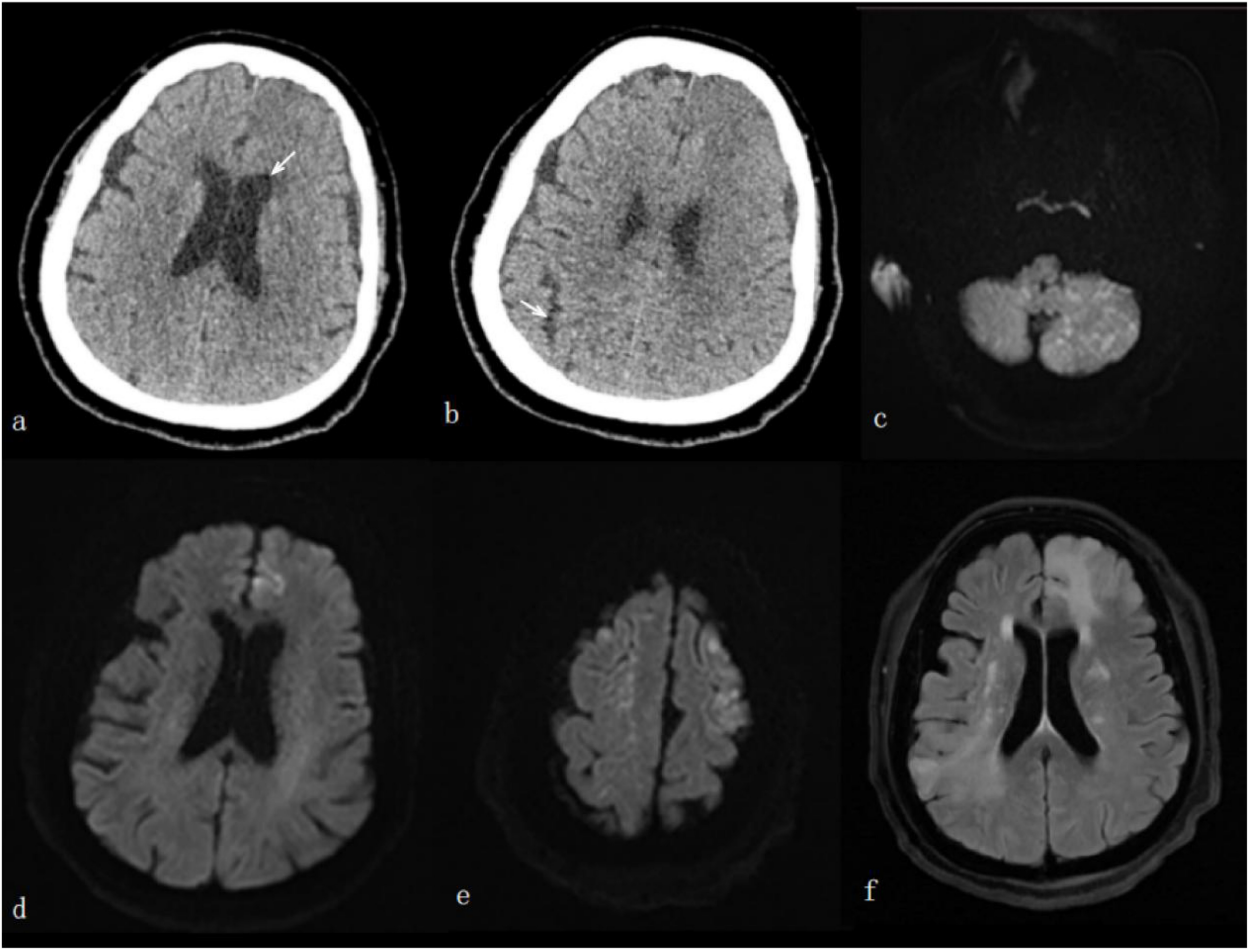
Head CT **(a,b)** obtained at 4 h after symptom onset demonstrated small air bubbles (arrows) in the anterior horn of the left lateral ventricle and the right cerebral sulci(arrows). Initial diffusion-weighted imaging (DWI) **(c–e)** showed multiple restricted diffusion lesions in the bilateral frontal-parietal lobes and cerebellar hemispheres; and follow-up brain FLAIR sequence **(f)** on the 9th day of hospitalization demonstrated that the lesions had fused and enlarged compared to the previous imaging.

Laboratory tests on admission revealed leukocytosis with a white blood cell count of 27.13 × 10^9^/L. Liver and renal function, coagulation profile, and D-dimer level were all within normal ranges. Four hours after symptom onset, we first performed non-contrast CT of the head and chest. The head CT demonstrated the presence of small air bubbles in the left frontal lobe and right temporal lobe (Figures 1a,b), and chest CT showed a focal lesion in the right upper lobe of the lung. Cranial magnetic resonance diffusion-weighted imaging (DWI) showed multiple diffusion-restricted lesions involving the frontal-parietal lobes and cerebellar hemispheres (Figures 1c–e), consistent with acute cerebral infarction. At 6 h following symptom onset, digital subtraction angiography (DSA) of the cerebral vasculature revealed no significant stenosis or occlusion of major intracranial arteries. On the 9th of hospitalization, follow-up cranial FLAIR sequence demonstrated that the infarcted lesions had fused and enlarged compared to prior imaging (Figure 1f).

### 2.3 Diagnosis and differential diagnosis

Given the patient’s acute onset of symptoms—sudden disturbance of consciousness and hemiplegia immediately following CT-guided percutaneous lung biopsy, combined with the presence of intracranial air bubbles on non-contrast head CT, punctate infarcts on DWI, and the absence of definite embolic occlusion of the great vessels on cerebral angiography, the diagnosis of cerebral infarction secondary to air embolism was established. The patient had no history of atrial fibrillation, and transthoracic echocardiography revealed no mural thrombus, effectively ruling out a cardioembolic source. Although she had conventional cerebrovascular risk factors such as hypertension and diabetes mellitus, DSA showed no significant stenosis or occlusion of major intracranial arteries, making it difficult to attribute the widespread and acute multifocal infarcts to a single large vessel atherosclerosis with stenosis.

### 2.4 Clinical course and management

Upon admission to our institution, her condition was critical. On day 1 at 18:00, mechanical ventilation was initiated, and the patient received 100% oxygen therapy for 4 h. However, the patient remained comatose, and physical examination was unchanged from admission. Given that prolonged administration of 100% oxygen is inadvisable, we gradually transitioned to low-flow oxygen via endotracheal tube while maintaining oxygen saturation above 94%. Concurrent anti-infective therapy was administered, initially with piperacillin–tazobactam, and subsequently switched to meropenem and cefoperazone–sulbactam based on sputum culture results. On hospital day 8, tracheostomy was performed to facilitate airway clearance. At 4:40 a.m. on hospital day 2, the patient developed status epilepticus, which was eventually controlled after combined therapy with multiple antiseizure medications, including continuous infusion of sodium valproate, intravenous bolus of diazepam 10 mg, intramuscular injection of phenobarbital sodium 0.1 g, and intravenous drip of lacosamide 0.2 g. The regimen was subsequently transitioned to oral lacosamide and levetiracetam.

Throughout the hospitalization, hemodynamic management was maintained with a target mean arterial pressure above 85 mmHg to ensure adequate cerebral perfusion. As the patient remained hemodynamically stable, the use of vasoactive agents was not indicated. Meanwhile, mannitol was administered intravenously (initially 150 mL every 6 h, subsequently tapered) for reduction of intracranial pressure (ICP). Additional comprehensive supportive care comprised anticoagulation, correction of acidosis, lipid-lowering therapy, nutritional support, and hypothermic therapy with an ice cap.

**FIGURE 2:**
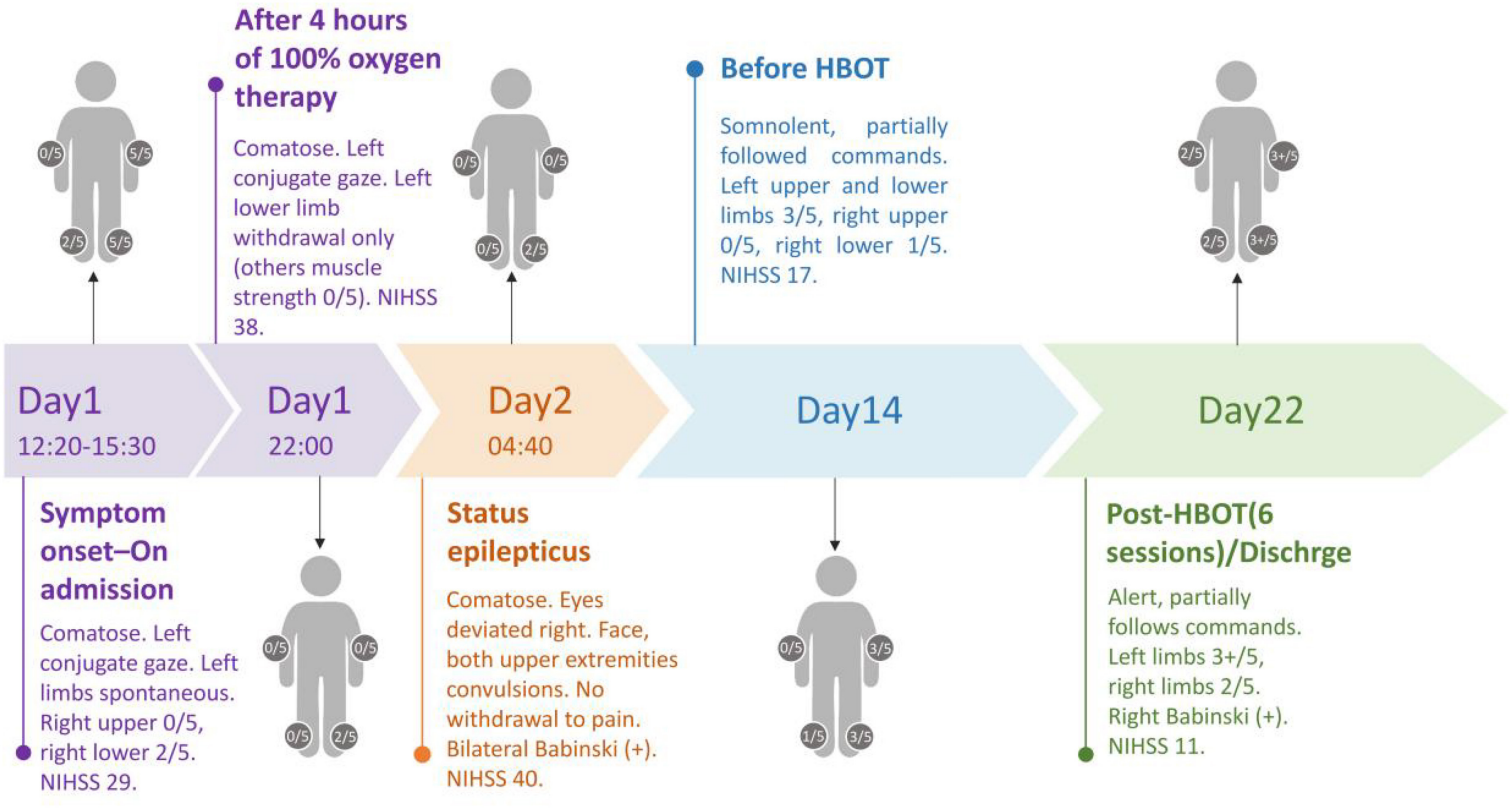
Clinical timeline from symptom onset to discharge.

The patient’s condition gradually stabilized, and hyperbaric oxygen therapy (HBOT) was initiated on day 14. The delay was primarily due to two factors. First, the patient’s grave condition during the first 2 weeks—including status epilepticus, fever, and the peak of cerebral edema—posed safety concerns for HBOT. Second, during the hospitalization, a biopsy report from another institution confirmed a diagnosis of lung adenocarcinoma. The family was informed that HBOT might potentially accelerate tumor progression, and they declined this intervention at the outset. It was not until infection was controlled and the patient’s neurological status improved from coma to somnolence that the family consented to HBOT. At 15 h following the first HBOT, the patient’s level of consciousness improved from drowsiness to alertness, with muscle strength in the left limbs showing slight amelioration compared with prior status. After six sessions of HBOT, the patient was alert and able to partially follow commands. Muscle strength in the left limbs improved to grade 3+/5, and in the right limbs to grade 2/5. The patient remained tracheostomy-dependent. No further seizures were observed, and vital signs were stable. The patient’s treatment process from day 1 to day 22 is shown in Figure 2. Ultimately, she was subsequently transferred to a rehabilitation hospital for further treatment.

## 3 Discussion

Air embolism is a pathological condition where air enters the circulatory system, forming emboli that obstruct blood vessels, leading to ischemia or infarction of distal tissues. In percutaneous lung biopsy, the incidence of symptomatic air embolism is approximately 0.08%. However, if systematic postoperative CT screening is performed, the total detection rate, including asymptomatic cases, can reach up to 3.8% (4, 5). Cerebral air embolism is a severe subtype of air embolism, referring to gas emboli entering the cerebral arterial system and triggering an acute ischemic stroke. Clinically, it often presents as sudden disturbances of consciousness, hemiplegia, aphasia, or seizures, constituting a clinical emergency with potentially fatal risks (6, 7). Iatrogenic cerebral air embolism has an acute onset, with clinical symptoms typically appearing during or within minutes to hours after an interventional procedure (3). The symptoms observed in this patient are consistent with the typical presentation of this complication.

CT-guided percutaneous lung biopsy is a clinically common and significant precipitating factor for cerebral air embolism (4). The pathogenesis involves three main mechanisms: ➀Direct pathway: The biopsy needle directly punctures a pulmonary vein under which air enters the pulmonary vein. ➁Pulmonary artery-pulmonary vein pathway: Air passes through the punctured pulmonary artery and enters the pulmonary venous system via the pulmonary capillary network or potential arteriovenous communications. ➂Bronchovenous fistula: The biopsy needle simultaneously traverses an airway (bronchus or alveolus) and an adjacent pulmonary vein, forming an abnormal fistula. When the alveolar pressure exceeds the venous pressure (such as during coughing, positive-pressure ventilation, or in the presence of emphysema), alveolar gas can enter the venous system through this fistula (6). Gas entering the pulmonary veins flows back to the left atrium and left ventricle and is eventually pumped into the systemic arterial circulation, leading to an arterial air embolism (8). Emboli ejected from the aortic arch are more likely to enter the brachiocephalic trunk and common carotid arteries, preferentially reaching the cerebral circulation. This can result in intracranial artery embolism and cause acute neurological deficits (9).

More revealing are the dynamic changes observed on the patient’s MRI, which elucidate the dynamic progression of neurological injury following cerebral air embolism. On the day of admission, DWI of the brain revealed “multiple indistinct patchy and small dot-like hyperintensities in the bilateral frontal-parietal lobes and bilateral cerebellar hemispheres,” with “more pronounced diffusion-restricted lesions in the left frontal-parietal lobes compared to the right” (Figures 1c–e). This presentation aligns with the early radiological characteristics of cerebral arterial air embolism: a multifocal, bilateral distribution primarily involving the middle cerebral artery territories (frontal-parietal lobes) and the posterior circulation (cerebellar hemispheres). The underlying mechanism is that gas emboli entering the systemic circulation, being light and highly mobile, tend to travel upstream with blood flow. Due to buoyancy, they are more likely to embolize the terminal branches of higher-position arteries, particularly the cortical branches of the anterior and middle cerebral arteries, as well as the superior cerebellar artery (10–13). The predominance of lesions on the left side may be attributed to hemodynamic factors or the anatomical course of the left common carotid artery, causing more emboli to enter the left internal carotid artery system after entering the aortic arch (14). A follow-up cranial MRI on the 9th day of hospitalization showed “fusion and enlargement of the lesions compared to previous imaging (Figure 1f). This evolution suggests that intracranial ischemic damage in patients with cerebral air embolism may continue to progress during the subacute phase, resembling the radiological imaging patterns reported by Tsushima et al. (15). The underlying mechanisms are complex and may include: ➀Redistribution and re-embolization of residual air bubbles: Following the initial embolism, air retained within cardiac chambers or pulmonary veins may re-enter the systemic circulation as hemodynamics change, triggering new embolic events. ➁Progressive ischemia mediated by microbubbles: Microbubbles undetectable by imaging can persistently occlude microvessels, leading to a stepwise progression of ischemic injury in affected organs. ➂Reperfusion inflammatory response: Restoration of blood flow to the ischemic regions may activate inflammatory pathways, exacerbating local tissue damage. ➃Intravascular inflammation and endothelial dysfunction: The movement of air bubbles within blood vessels can directly damage endothelial cells, triggering inflammatory cell infiltration and vasoconstriction, thereby causing a sustained decrease in cerebral blood flow (15). This imaging evolution underscores the diagnostic value of the DWI sequence, which can clearly depict acute ischemic lesions at a very early stage and is the most sensitive imaging method for confirming cerebral air embolism (16). The radiographic progression observed in this case also suggests that for patients with a high clinical suspicion of cerebral air embolism, even if the early DWI only shows atypical, small dot-like lesions, these should be regarded as positive findings. Clinicians should remain vigilant for the potential fusion and enlargement of lesions in subsequent stages.

The diagnosis of latrogenic cerebral air embolism is primarily based on a comprehensive assessment of interventional procedures history, typical clinical manifestations, and imaging evidence. Imaging studies are key to a definitive diagnosis: cranial CT can directly display pneumatosis (gas density) within the brain, though its detection rate is relatively low; the DWI sequence of cranial MRI is highly sensitive to acute ischemia, clearly showing multiple diffusion-restricted lesions in bilateral cerebral hemispheres or cerebellum, presenting a characteristic “multi-territorial” infarction pattern (17). It is noteworthy that early imaging examinations may show no abnormalities in some patients. Therefore, for individuals presenting with neurological symptoms following high-risk procedures, a high suspicion of cerebral air embolism should be maintained even if initial imaging results are negative, and short-term follow-up imaging should be considered if necessary (18). The initial cranial CT in this case revealed small air bubbles in the left frontal lobe and right temporal lobe, directly confirming that gas had entered the ventricular system and subarachnoid space. This finding serves as a significant direct sign of cerebral air embolism. It is important to emphasize that, although early CT may not show typical infarction foci, the detection of intracranial gas (whether in the brain parenchyma, ventricles, or subarachnoid space) holds crucial diagnostic value in itself.

As a clinical emergency, cerebral air embolism requires a comprehensive treatment protocol to be implemented immediately. Once suspected, the patient should be administered 100% pure oxygen without delay. This not only improves tissue oxygenation but, more importantly, establishes a nitrogen partial pressure gradient, promoting the dissolution and absorption of nitrogen in the bubbles, thereby buying time for subsequent treatment (19). Simultaneously, provided the patient’s vital signs are stable, a head-down position (Trendelenburg position) may be considered. This utilizes the buoyancy of the bubbles to trap them within the heart, reducing the risk of entry into the cerebral circulation (20). In addition, active fluid resuscitation, antiepileptic therapy, and symptomatic supportive measures such as dehydration to reduce intracranial pressure are crucial for stabilizing the patient’s condition (18). Among the comprehensive treatment strategies, HBOT plays a central role and is widely recognized as the first-line treatment (19). The timing of treatment is critical. Early administration of HBOT, particularly within 6–8 h of symptom onset, is significantly associated with favorable neurological outcomes (19). Even if symptoms show spontaneous remission, HBOT should be initiated as early as possible due to the risk of secondary deterioration (15).

Due to severe comorbidities, including pulmonary infection and generalized tonic-clonic seizures, the patient was confined to the intensive care unit and thus received 100% oxygen inhalation initially. HBOT was not initiated until day 14 of the illness. Although this timing was later than the conventional therapeutic window, the patient’s neurological function improved rapidly following HBOT. This not only confirms the efficacy of HBOT but also indicates its potential clinical benefit even when treatment is delayed.

In summary, although the incidence of symptomatic cerebral air embolism following percutaneous lung biopsy is rare, approximately one-third of such cases result in severe sequelae or death. Relevant Specialist must be familiar with the clinical and radiological presentations of iatrogenic cerebral air embolism as well as emergency medical interventions to reduce the likelihood of fatal complications.

## Data Availability

The original contributions presented in this study are included in this article/supplementary material, further inquiries can be directed to the corresponding authors.
